# A Structural Equation Modeling of the Relationships Between Parenting Styles, Students’ Personality Traits, and Students’ Achievement Goal Orientation

**DOI:** 10.3389/fpsyg.2021.805308

**Published:** 2022-01-11

**Authors:** Faramarz Asanjarani, Khadijeh Aghaei, Tahereh Fazaeli, Adnan Vaezi, Monika Szczygieł

**Affiliations:** ^1^Department of Counseling, Faculty of Education and Psychology, University of Isfahan, Isfahan, Iran; ^2^Department of Foreign Languages, Faculty of Humanities & Physical Education, Gonbad Kavous University, Gonbad Kavous, Iran; ^3^Department of Counseling, Islamic Azad University, Khomein, Iran; ^4^Department of Neurocognitive Psychology, Faculty of Pedagogy and Psychology, Institute of Psychology, Pedagogical University of Krakow, Kraków, Poland

**Keywords:** students’ achievement goal orientation, parenting styles, personality traits, structural equation modeling, psychoticism, extraversion

## Abstract

Recently, researchers have shown an increased interest in achievement goal orientation correlates. What is not yet clear is the detailed relationships among students’ goal orientation, students’ personality traits, and parenting style. In so doing, this research responds to the need to analyze the importance of parenting styles (permissive, authoritative, and authoritarian) and students’ traits (psychoticism, neuroticism, and extraversion) in explaining the achievement goal orientations (mastery approach, mastery avoidance, performance-approach, and performance-avoidance). In the exploratory correlational study, 586 Iranian students along with their parents were selected as the sample so as to evaluate the structure of the relationships between these variables. The results indicate that students’ psychoticism and neuroticism predict students’ goal orientations (positively: performance and mastery avoidance and negatively: mastery and performance approach) while extraversion did not. Only the authoritative style predicts mastery approach (positively) and psychoticism trait (negatively). Permissive and authoritarian styles do not directly or indirectly predict students’ goal orientations.

## Introduction

Acquiring a broader knowledge of students’ social and academic performance by looking into all aspects of students’ life is of significance. There is a considerable interest among educators and researchers to decode those factors contributing to students’ success and learning process (Kurmanov et al., 2015; Tondeur et al., 2016a,b; Conn, 2017). The better educators and professionals understand the various psychological and environmental factors that determine student success, the better they can help individuals. Studies on achievement goals have moved on in several directions, including revising the underlying frameworks for achievement goals and consequently their implementation in educational settings for a wide range of populations (Elliot et al., 2011). Current studies accentuate that parenting styles (Spera, 2005; Chen, 2015; Diaconu-Gherasim and Măirean, 2016) and the personality traits (Muenks et al., 2017; Sorić et al., 2017) as two potential factors affecting achievement goals orientations. Recent literature (Lochbaum et al., 2017) indicate that the 2 × 2 achievement goals were significantly correlated amongst each, but only a few of the relationships were medium in meaningfulness. Most relationships were small in meaningfulness. Given the results of previous studies, investigating personality traits, parenting styles, and achievement orientation goals in association with one another as an unaddressed issue in the literature may add insight into our knowledge of students in academic settings.

## Achievement Goals in Academic Settings

Elliot et al. (2011) have defined achievement goals as competency-based goals used to direct behaviors of the individuals. In other words, achievement goal orientations are the cognitive representations of students’ purpose to get engaged in an academic task. Achievement goal orientations were initially introduced in the late 1970s and early 1980s following Dweck and colleagues’ research (Maltais et al., 2015). Achievement goals include two main types, (1) mastery goals which encompass attaining mastery standards and developing competence, and (2) performance goals (Linnenbrink-Garcia and Barger, 2014). Besides, in a trichotomous achievement goal framework, which Elliot and Harackiewicz (1996) introduced, performance goals consist of two concepts, performance-approach, also known as outperforming, and performance-avoidance which refer to not performing poorly relative to others.

As Elliot and Murayama (2008) argue, an essential characteristic of adolescence, which determines their educational competence, is achievement goal orientations. Elliot and Murayama (2008) suggest that achievement goals include four categories: (1) mastery-approach in which the goal is defined as attaining task-based or intrapersonal competence; (2) performance-approach in which the goal focuses on attaining normative competence; (3) mastery-avoidance in which the goal focuses on avoiding task-based or intrapersonal incompetence; and finally, (4) performance-avoidance in which the goal is centered on avoiding normative incompetence (Elliot and Murayama, 2008).

For the last three decades, the achievement goals have been an underlying construct in achievement studies. Various conceptual models of achievement goals have been introduced in this timespan: dichotomous, trichotomous, 2 × 2 (Elliot, 2005), and a more recent version, the 3 × 2 conceptual frameworks (Elliot et al., 2011). Correspondingly, many measures and scales make the measurement of achievement goals possible (Nicholls et al., 1985; Elliot and Church, 1997; Elliot and McGregor, 1999). In a recent meta-analysis, Huang (2011) reported that the most frequently used tools to measure achievement goals was the Achievement Goals Questionnaire (AGQ), developed by Elliot and McGregor (1999), and an improved version, Achievement Goal Questionnaire-Revised (AGQ-R) introduced by Elliot and Murayama (2008). Furthermore, the Achievement Goals Questionnaire is the most frequently used tool to measure the 2 × 2 achievement model.

A few studies have investigated how students’ goal orientations are associated with their personality, parenting style, and academic competency. Similar to the findings conducted in Western and European contexts, studies in Iran accordingly confirm that goal orientations are correlated with academic achievement (Dehghani Nazhvani and Zarepour, 2016), academic motivation (Komarraju et al., 2009), perceived classroom goal structures, cognitive and metacognitive strategies (Sungur and Güngören, 2009).

## Achievement Goals and Parenting Styles

The possible effect of parenting behaviors and parenting styles on the development of the academic performance in students has been theoretically supported (Turner et al., 2009; Chen, 2015; Masud et al., 2016; Carlo et al., 2018). As an example, Pinquart (2016) points out that parental responsiveness (i.e., warmth), autonomy granting, behavioral control, and authoritative parenting style were correlated with better academic performance in students. Besides, unpleasant parental control, psychological authority, and abusive, authoritarian, and permissive parenting styles were associated with lower achievement in students. Pinquart (2016) also indicates that parenting dimensions and styles also predicted academic achievement change over time. Pinquart (2016) also theorized that child age and ethnicity might be the moderating effects of academic achievement and quality of parenting behaviors.

Based on Baumrind (1978) typology of parenting behavior, parenting styles can be characterized in two dimensions: (1) demandingness—the extent to which parents demonstrate control, demands for maturation, and supervision, and (2) responsiveness—the extent based on which they display affective warmth, acceptance, and involvement toward their children. Baumrind (1991) argues that based on these two dimensions, parenting behavior includes three styles: authoritarian, authoritative, and permissive. In a newer extension of the model’s Maccoby (1984) divided the permissive style to create a fourth style: neglectful (also sometimes termed “uninvolved”).

In Baumrind (1991)’s conceptualization of parenting styles, it is suggested that those parents with authoritarian parenting styles demonstrate high levels of demandingness, along with low levels of responsiveness. These parents primarily focus on controlling their children. In contrast, Baumrind (1991) described authoritative parents as those parents with both higher levels of demandingness and higher levels of responsiveness. Even though these parents observe their children’s behavior, they use no punitive forms of control when guidelines for behavior are not met. Authoritative parents appreciate their children’s points of view and support their children. The third parenting style (i.e., permissive) is characterized by low levels of demandingness and high levels of responsiveness. Similar to the authoritative parenting style, parents show a warm and accepting attitude toward their children. Notably, in this parenting style few regulations are implemented and there exists no control over their children because of their no demanding behaviors (Baumrind, 1991). In the fourth and the final parenting styles, neglectful/uninvolved parents are distinguished by low levels of both demandingness and responsiveness. A neglectful/uninvolved parenting style does not provide their children with any support or attention. They do not seek to direct their children’s behaviors although they remain rather unconcerned about their children’s lives (Maccoby, 1983).

Parenting styles based on Baumrind (1991) typology, particularly authoritative and authoritarian parenting styles, were reported to be correlated with Iranian students’ academic achievement (Besharat et al., 2011; Dehyadegary et al., 2012). Considering the positive values of authoritative parenting, they also demonstrated that parenting style contributes to a more significant motivation and academic performance among Iranian students.

Several studies have found a positive relationship between authoritarian parenting styles and student achievement (Baumrind, 1991). Baumrind (1967) was one of the first studies to report on this relationship. As a result of a longitudinal sample of preschool to teenage children, Baumrind found that preschoolers of authoritarian parents were more mature, independent, prosocial, active, and successful-oriented than other children. On the other hand, preschoolers of permissive parents scored lowest on autonomy, self-control, and competence measures (Baumrind, 1989).

Following Baumrind’s early work, Dornbusch, Steinberg, and their colleagues conducted a series of studies to explore the influence of parenting styles on adolescent success. These studies used data from large-scale surveys of more than 6,000 adolescents in Wisconsin and California. One of the first studies in this series found that parents who displayed higher levels of parental authority by providing their children with high demands for warmth, autonomy, and maturity had children with higher levels of achievement (Steinberg et al., 1989). In another study, Steinberg et al. (1992) found that authoritarian parenting was associated with adolescent academic engagement. These findings have led researchers to wonder why authoritarian parenting styles are associated with positive academic outcomes.

Despite this, very few studies have investigated the correlation between goal orientation and parenting styles (Gonzalez et al., 2001, 2002; Huang, 2011). These researchers noted that perceived authoritative parenting styles were closely correlated with the mastery goal orientation while perceived authoritarian parenting styles were positively associated with the performance goal orientation in both high school and undergraduate students. With reporting similar findings, Chen (2015) also examined the relationship between parenting styles and students’ goal orientations among Hong Kong students.

## Achievement Goals and Personality

Eysenck’s questionnaires are well-known in personality psychology. Both adult and junior forms are still widely used for clinical, scientific, and professional purposes. Eysenck’s instruments have been refined based on the received contributions from many experts. Research efforts are made so as to improve the psychometric properties of the instruments and enrich their underlying theories. The last revision of the adult form evaluated four PEN-L characteristics (Psychoticism, Extraversion, Neuroticism, Lie) with 100 items in contrast with the earlier form, including 89 ones (Corulla, 1990; Colledani et al., 2018a,b).

Despite the extensive literature on achievement goals and academic performance, there exists a paucity of research to investigate the correlation of personality with students’ achievement goals. In the last four decades, the research literature considered extraversion as the most crucial dimension of personality (Digman, 1990). Individuals with a high level of extraversion are characterized as assertive, talkative, energetic, and active (Lucas et al., 2000) and a considerable level of positive attitudes (Lucas and Baird, 2004). It has even been hypothesized that extraversion gives happiness to individuals and a positive attitude toward the environment (Pavot et al., 1990; Pishva et al., 2011).

Research literature has also noted a relationship between certain personality traits and achievement goal orientations. Judge et al. (2002) in a meta-analysis contended that neuroticism and conscientiousness exhibit a significant direct correlation with performance goal orientation. In keeping with, Payne et al. (2007) found that conscientiousness, extraversion, and openness are positively associated with learning (i.e., mastery) goal orientation. Rather, neuroticism and introversion often predict performance-avoidance goal orientation.

Results reported in case studies are occasionally inconsistent. These inconsistencies are rooted in the conceptualizations and instruments used to measure the goal orientations variable. For example, Colquitt and Simmering (1998) found that conscientiousness was negatively correlated with a performance orientation. In contrast, Zweig and Webster (2004) found that conscientiousness (among several other traits) was positively associated with the mastery and performance-approach goal orientation although negatively correlated with the performance-avoidance goal orientation. On the other hand, neuroticism was negatively associated with mastery goal orientation though positively related to performance-approach and performance-avoidance goal orientations (Zweig and Webster, 2004). Vermetten et al. (2001) also suggested that conscientiousness and agreeableness are related to goal orientation.

## Present Study

**FIGURE 1:**
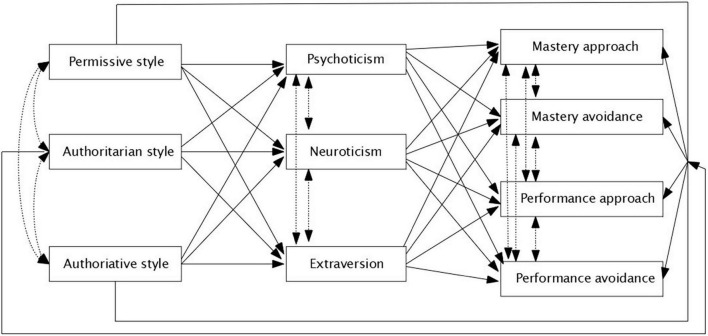
Theoretical tested model on dimensions of parenting styles, personality traits, and achievement goal.

Despite the collection of studies in this field, to the best of researchers’ knowledge, there is no evidence focuses on the relationships among parenting styles (permissive, authoritative, authoritarian), students’ personality traits (psychoticism, neuroticism, extraversion), and students’ achievement goal orientation (mastery approach, mastery avoidance, performance approach, performance-avoidance). As an effort to fill this research gap, the researchers adopt an exploratory approach to data analysis to test how each parenting style and personality traits contribute to students’ achievement goal orientations. In line with the main objective of the current study, the correlations between the variables and the structure of the relationships among indicators are addressed to test the theoretical structural model, which assumes such detailed relationships (see Figure 1).

Therefore, the following research questions were formulated

• Do parental styles determine students’ personality traits and achievement goals orientation?
• Do students’ personality traits determine their achievement goal orientation?

Our theoretical model assumes that various dimensions of parental styles directly and indirectly (*via* students’ personality traits) predict those of students’ goal orientation. It is because the personality traits of children depend on parenting styles (Weiss and Schwarz, 1996). We also assume that students’ personality traits predict various types of students’ goal orientation. Defining structural model provides us with the indices of parental styles, students’ personality traits, and students’ goal orientation which are correlated with each other (Gonzalez et al., 2002; Rivers, 2008; Jensen, 2015; Kosterelioglu, 2018).

## Materials and Methods

### Samples and Subjects

The sample of this study consisted of 625 Iranian students (boys *N* = 339, aged *M* = 15.15, *SD* = 0.55, range 14–17; girls *N* = 286, aged *M* = 15.15, *SD* = 0.57, range 14–16). Using convenience sampling, participants were selected from 16 schools in Tehran, Iran. Students were all Muslims, and Farsi was their mother tongue. To have a representative sample, students were recruited from different school types and regions. Students’ parents were also recruited (*N* = 586): fathers *N* = 120, aged *M* = 47.79, *SD* = 6.19, range 38–66; mothers *N* = 466, aged *M* = 41.77, *SD* = 4.92, range 27–58.

Few questionnaires (<10 ones) were incomplete or eligible, therefore, they were excluded in data analysis.

## Measurements

### Achievement Goal Orientations

A 12-item Achievement Goal Questionnaire (AGQ) (Elliot and McGregor, 1999; Hejazi et al., 2012) was applied. The scale consists of four subscales, each of which with three items: mastery approach (e.g., “I want to learn as much as possible from this class”), mastery avoidance (e.g., “I worry that I may not learn all that I possibly could in this class”), performance-approach (e.g., “It is important for me to do better than other students”), performance-avoidance (e.g., “I just want to avoid doing poorly in this class.”). Students were asked to respond on a Likert-type scale on to what extent they agree with each statement (1 = strongly disagree to 5 = strongly agree). The higher score in each subscale, the higher intensity of that goal orientation. Hejazi et al. (2012) evaluated the psychometric properties of the questionnaire. They showed that the α for the four subscales were as follows: mastery-approach goals (α = 0.61); mastery-avoidance goals (α = 0.62); performance-approach goals (α = 0.61) and performance-avoidance goals (α = 0.48). Low reliability of the AGQ is due to a small number of items.

### Personality Traits

As for personality traits, an 81-items Junior Eysenck Personality Questionnaire (JEPQ) (Eysenck and Levey, 1972; Asgari, 2005) was used to evaluate three dimensions of students’ personality: Psychoticism (P), Extraversion (E), and Neuroticism (N). In addition, the questionnaire includes a 20-item Lie (L) scale, which is a measure of social desirability (we did not use this subscale in the analysis). This questionnaire was scored on a three-point scale (“Yes”/“Don’t know”/“No”; “Don’t know” responses were recorded as missing data). The higher sum of points, the more intense each trait. The high reliability and good validity of JEPQ have been well-established in the Iranian sample (Asgari, 2005) which showed that the α for the three subscales are as follows: Psychoticism (α = 0.72), Extraversion (α = 0.66), and Neuroticism (α = 0.78).

### Parenting Styles

In this study, Baumrind’s parenting styles questionnaire developed by Baumrind (1972) and translated by Hosseinpour (2002) was used. It comprised 30 items and three dimensions: permissive style, authoritarian style, and authoritative style. The response pattern to the questions was a 5- point Likert scale scored from 0 to 4. The higher sum of points, the greater degree of use of a given style.

Buri (1991) estimated the reliability of this questionnaire based on the test-retest method as 0.81 for permissive, 0.92 for authoritarian and 0.92 for authoritative parenting styles. In assessing the validity of this instrument, he indicated the relationship between permissible and authoritarian -0.50 and between authoritative and authoritarian -0.52. In Iran, Mahdavi et al. (2013) has given the reliability of subscales by retest tests, 0.69 for the allowable style, 0.77 for the authoritarian, and 0.73 for the authoritative.

## Procedure

The procedure of this study includes the resulting steps: Following obtaining ethical approval from the Beauro of Education (ethical code no: 1400/114854), students were recruited using convenience sampling. Since Iranian schooling systems are gender- segregated, we thus recruited the same number of schools for boys and girls to have access to both genders. Data were collected by the students and their parents during the school meetings. Each questionnaire of students and parents was connected with a confidential number. Students and mothers were assured about the confidentiality of the results, the possibility of voluntary participation in the study, and the possibility of withdrawing from participation at any time. Students completed AGQ, JEPQ, and answered demographic questions. Meanwhile, parents also filled out parenting questionnaire and answered the related demographic questions. In order to fill in the questionnaires, 30 and 20 min were allotted to the students and the parents respectively. Then, the incomplete or non-eligible questionnaires were excluded in data analysis Notably, fathers in Iranian educational settings are rarely engaged. Rather, mothers play more crucial role in this regard. Given the cultural factor, the sample in this study composed of relatively fewer fathers than mothers.

## Results

Descriptive statistics and Pearson’s correlations matrix were calculated in Statistica 13 and are presented in Table 1. We interpreted effect size in the following way: *r* < 0.20 very weak, 0.20–39 weak, 0.40–0.59 moderate, 0.60–0.79 strong, and >0.80 very strong (Evans, 1996).

**TABLE 1:** Descriptive statistics and Pearson’s correlation matrix.

		*M*	95% *CI*	α	*SD*	1	2	3	4	5	6	7	8	9
1	Mastery approach	3.55	3.50–3.62	0.59	0.81									
2	Mastery avoidance	2.83	2.76–2.93	0.71	1.09	−0.36***								
3	Performance approach	4.09	4.04–4.16	0.35	0.80	0.28***	−0.19***							
4	Performance avoidance	3.13	3.05–3.18	0.74	0.78	0.03	0.21***	0.20***						
5	Permissive style	2.49	2.45–2.53	0.63	0.47	0.02	0.08	–0.08	0.09*					
6	Authoritarian style	2.52	2.48–2.57	0.72	0.55	–0.05	0.11**	–0.02	0.04	0.11*				
7	Authoritative style	4.00	3.96–4.05	0.75	0.51	0.12**	–0.07	0.06	–0.01	–0.05	−0.25***			
8	Psychoticism	0.26	0.25–0.27	0.63	0.15	−0.17***	0.29***	−0.16***	0.08	0.10*	0.11**	−0.13**		
9	Neuroticism	0.52	0.50–0.54	0.81	0.21	−0.20***	0.36***	–0.06	0.28***	0.06	0.06	0.06	0.31***	
10	Extraversion	0.73	0.72–0.74	0.68	0.15	0.09*	–0.04	0.07	–0.02	0.02	–0.07	0.06	−0.12**	−0.21***

Pairwise deletion was applied in the case of missing data; N = 556. *p < 0.05, **p < 0.01, ***p < 0.001. α, Cronbach’s alpha.

First, we tested correlates (personality and parenting styles) of achievement goal orientation. The results indicated that mastery approach orientations were positively and very weakly related with authoritative style and extraversion and negatively and very weakly with psychoticism and neuroticism. Mastery avoidance orientation was positively and very weakly related with authoritarian style and positively and weakly with psychoticism and neuroticism. The performance approach correlated very weakly and negatively only with psychoticism. Finally, performance-avoidance orientation correlated positively and very weakly with permissive style and positively and weakly with neuroticism.

In the next step, whether variable indicators are correlated with each other or not were examined. The results are as follows. Achievement goal orientations were mostly correlated weakly and negatively or positively with each other. Permissive style was correlated with the authoritarian style positively and very weakly; the authoritative style was negatively and weakly related with authoritarian style. Psychoticism was positively and moderately related with neuroticism, and extraversion was very weakly/weakly and negatively related with psychoticism and neuroticism, respectively.

**FIGURE 2:**
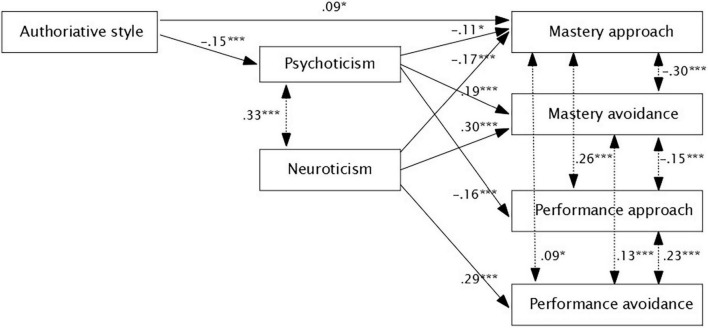
Obtained empirical model. *,***Represents level of significance.

Finally, we tested the theoretical model (see Figure 1) using a structural equation model in *lavaan* (Rosseel, 2012). We used the Diagonally Weighted Least Squares estimator (Mindrila, 2010). We evaluated the adequacy of the model using the following indices: non-significant *p*-value in χ^2^test, the root mean square error of approximation (RMSEA) and the standardized root mean squared residual (SRMR) smaller than 0.08, the comparative fit index (CFI), and the Tucker-Lewis index (TLI) above 0.95. The theoretical model was modified to fit the data well. We deleted the non-significant path, and we kept only the significant path. The final structural model (see Figure 2) was very well-fitted to the data: $\chi_{\left(6\right)}^{2}=$ 5.41, *p* = 0.49, CFI = 1, TLI = 1, RMSEA [90% *CI*] = 0 [0, 0.05], SRMR = 0.016.

The obtained empirical model presents that mother’s authoritative style directly determines students’ psychoticism and mastery approach. Psychoticism is related with neuroticism. Both personality traits predict chosen students’ achievement goal orientation. Dimensions of achievement goal orientation are related each other. Standardized regression coefficient is presented next to the arrows.

More precisely, it can be stated that the theoretical and empirical model differ in many points. Indeed, the results indicated that mastery approach orientation is very weakly/weakly and negatively predicted by psychoticism and neuroticism, respectively, and very weakly and positively by authoritative parenting style. Mastery avoidance is predicted weakly and positively by psychoticism and neuroticism. The performance approach is predicted weakly and negatively by aggression, while performance-avoidance is predicted weakly and positively by neuroticism. Extraversion was not related to students’ goal orientations. Also, authoritarian and permissive styles did not predict directly or indirectly students’ goal orientations. Authoritative style additionally predicted weakly and negatively psychoticism and psychoticism was negatively and weakly related to neuroticism. The empirical model indicated that students’ goal orientations are mostly related to each other.

## Discussion and Conclusion

The results of the study showed that students’ psychoticism and neuroticism very weakly or poorly predicted students’ goal orientation (positively: performance and avoidance of mastery, and negatively: mastery and performance approach) whereas extroversion did not. Only authoritative style predicted mastery approach (very weak and positive) and predicted psychoticism negatively and weakly. Permissive and authoritarian styles did not directly or indirectly predict students’ goal orientation. The findings of the current study are consistent with previous studies (Ginsburg and Bronstein, 1993; Gonzalez et al., 2001, 2002; Komarraju et al., 2009; Clark and Schroth, 2010).

Previous studies have demonstrated that older students tend to have lower levels of performance-approach (Burley et al., 1999). Komarraju et al. (2009) demonstrated that extraversion was positively associated with extrinsic motivation. Furthermore, Thiele et al. (2018) introduced a model which indicated that extraversion is positively related to performance and the two constructs are conceptually related. Therefore, higher degrees of extraversion could plausibly contribute to higher goal achievement orientations or vice versa.

This study showed that authoritative style predicted mastery approach. Students who adopted a mastery-approach orientation seek to achieve comprehension and learning objectives (Harackiewicz et al., 1998; Pintrich, 1999). Those who adopted this type of goal orientation were keen to try and increased their understanding and skills by learning as much as possible. Students with an objective avoidance mastery orientation focused on avoiding misunderstandings. In addition, students who adopted this type of goal orientation seek to learn in order to avoid a lack of mastery or forgetting what they have learned. They strived to avoid losing out on a task or losing their skills, abilities or knowledge (Elliot and McGregor, 2001).

In the present study, it was found that parents who use an authoritarian style are likely to tell their children that they must do well in school and associate doing well with getting good grades. That maybe why students resort to what their parents ask them to do and focus on performing better than others in order to get better grades. The present study makes a contribution to the literature by focusing only on Iranian students in order to add to what is known about the differences between cultural groups regarding the use of parenting styles as a predictor of student performance.

Some studies indicated that avoidance motivation was positively related to neuroticism (Payne et al., 2007; Komarraju et al., 2009). Additionally, students with lasses-fair or permissive parenting reported a significant focus on performance-avoidance goals (Gonzalez and Wolters, 2006). Some researchers also reported higher associations between goal orientations and parenting styles or personality traits (Gonzalez and Wolters, 2006; Payne et al., 2007; Komarraju et al., 2009). In contrast, some other studies showed no significant association between the studied variables. For instance, Miller and Speirs Neumeister (2017) indicated that personality traits and parenting styles did not have any significant relationship with performance goal orientation. One explanation for inconsistent findings can be different possible variables that are not included in the present study although presented in the literature for the origin and development of goal orientations (Kaplan and Maehr, 2007). Students’ adoption of mastery goals (Roeser et al., 1996), use of effective learning strategies (Kaplan and Maehr, 2007; Maltais et al., 2015), and attitudes toward school and class (Kaplan and Maehr, 2007) were found to be associated with mastery goal as well.

Research literature has so far addressed the relations between students’ personality and goal achievement orientation (Alhadabi and Karpinski, 2020; Jones et al., 2021). However, the current study addressed this topic on a large scale, with a representative sample of over 500 adolescents through a design that included questionnaires administered for both parents and their children.

## Limitations

There are some limitations in the present study that should be pointed out. First, the correlational design used in this study may prevent drawing specific conclusions. As an example, social desirability may have prevented some students from objectively assessing achievement goals or personality. This study might also suffer from the limitation of paper-pencil measurement tools and self-report instruments. Although this type of research has the advantage of increased sample size and ease of data collection in educational studies, there is always the issue of objectivity. However, most studies looking at self-reports of students in higher education state that the studies’ self-reports and actual abilities of the students are positively correlated (Hayek et al., 2002). Considering the characteristics of the population, we used convenience sampling, which might have affected the data collection result or procedures. As mentioned earlier, many other variables, such as self-schemas, situation-schemas, values, or needs, are presented in the literature to be associated with goal orientations (Kaplan and Maehr, 2007). It is recommended that future studies include these variables in the model. Finally, it is not common for fathers to be engaged in educational issues of their children. Considering this cultural factor, the number of fathers is relatively fewer than mothers. Another limitation of the study that might affect the result of the research is the low reliability of the questionnaire used in our study.

## Implications and Future Trends

The findings of the present study have significant implications for school curricula, parent education and community outreach programs aim to reflect the importance of parenting styles. Besides, some studies also implied that parenting style can impact students who are performing badly at school (see, for example, De Silva et al., 2018; Asanjarani et al., 2021). They showed that lower levels of parental involvement and expectations that students receive from their parents may lower motivation (Mitchall and Jaeger, 2018).

Moreover, it is claimed that four parenting styles can clearly be differentiated based on two dimensions of parental acceptance and parental control. Hence, the study can be of value for further research led to explore the perception of parental acceptance and control, especially the latter, might be different among adolescents.

Specifically, in the present study, we only relied on parenting styles reported by the parents, and we did not investigate the perceived parenting styles reported by adolescents. Future studies may benefit from this issue as well. In addition, increasing parents’ awareness on this issue can be more contributive.

It is recommended that the parent education program address this issue by encouraging higher academic supervision and parental academic involvement. In addition, school administrators need to work closely with parents to encourage parental involvement and address critical issues such as lack of parenting knowledge.

Finally, the other practical implication is that teachers and guidance counselors should be aware of the various parenting practices and cultural differences that students bring to the school environment. When guiding students, school administrators, especially counselors, should consider a student’s post-high school aspirations or plans, as well as personal and family goals.

## Data Availability Statement

The raw data supporting the conclusions of this article will be made available by the authors, without undue reservation.

## Ethics Statement

This study was reviewed and approved by the Markazi Province Bureau of Education (ethical code no: 1400/114854). Written informed consent to participate in this study was provided by the participants’ legal guardian/next of kin.

## Author Contributions

All authors in the present study equally reviewed, edited, and revised the various sections of the manuscript for important intellectual content, contributed to the interpretation of the results and the final manuscript, and approved the submitted version.

## Conflict of Interest

The authors declare that the research was conducted in the absence of any commercial or financial relationships that could be construed as a potential conflict of interest.

## Publisher’s Note

All claims expressed in this article are solely those of the authors and do not necessarily represent those of their affiliated organizations, or those of the publisher, the editors and the reviewers. Any product that may be evaluated in this article, or claim that may be made by its manufacturer, is not guaranteed or endorsed by the publisher.
